# Comparison of treatment outcomes of stable and unstable developmental dysplasia of the hip with the Tübingen splint

**DOI:** 10.3389/fped.2022.976367

**Published:** 2022-08-25

**Authors:** Emmelie Chaibi, Claire-Anne Saugy, Eleftheria Samara, Pierre-Yves Zambelli, Sophie Rosa Merckaert

**Affiliations:** Unit of Pediatric Orthopedics, Department of Women – Mother – Child’s Care, Centre Hospitalier Universitaire Vaudois, Lausanne, Switzerland

**Keywords:** DDH, Graf method, spica cast, closed reduction, Tübingen splint, hip ultrasound

## Abstract

**Background:**

The Tübingen splint was initially developed for the treatment of stable developmental hip dysplasia (DDH). Later on, some authors expanded its include for the treatment of unstable DDH, but there remain some controversies in the literature. This study aims to compare the outcome between stable and unstable DDH treated with a Tübingen splint.

**Methods:**

Epidemiological data and ultrasonographic data of all infants diagnosed with DDH and initially treated with a Tübingen splint at our institution between May 2017 and February 2020 were assessed retrospectively. We divided the population into stable and unstable hips using the Graf classification. Age at treatment initiation, duration of treatment, complications, and radiological outcome between 12 and 24 months were investigated.

**Results:**

We included a total of 45 patients (57 hips) affected by DDH treated with the Tübingen splint. Treatment has been successful in 93% of stable hips and only 40% of unstable hips. Radiological outcome at 1-year follow-up significantly correlated with initial Graf classification (*p* < 0.001).

**Conclusion:**

The Tübingen splint is a safe and effective treatment for stable hips, nevertheless, for unstable hips, closed reduction, and spica cast remains the gold standard.

## Introduction

Developmental dysplasia of the hip (DDH) is defined as insufficient acetabular coverage of the femoral head and can range from mild dysplasia to total dislocation of the joint (1, 2). Incidence of DDH ranges between 1 and 20 per 1,000 infants per year depending on the literature and region (3). Ultrasonography according to Graf is the method of choice for diagnosis within the first months of life (4, 5). The Graf method allows us to classify DDH according to its severity into ultrasound stable (hip types I–IIc) and unstable hips (hip type D to IV) (6) (Table 1). As type IIc hip can be stable or unstable, stress examination needs to be carried out to confirm a stable or unstable joint.

**TABLE 1:** The Graf classification system of developmental dysplasia of the hip (19).

Type	Age	Alpha angle (°)	Beta angle (°)	Description	Stability
I	Any	>60	<55	Normal	Stable
IIa^+^	0–6 weeks	50–59	55–77	Immature hip	Stable
IIa^–^	6–12 weeks	50–59	55–77	Severe immature hip	Stable
IIb	>12 weeks	50–59	55–77	Dysplastic hip	Stable
IIc	Any	43–49	<77	Dysplastic hip	Stable
D	Any	43–49	>77	Everted labrum	Unstable
III	Any	<43	–	Dislocated	Unstable
IV	Any	Not measurable	–	Dislocated with labrum interposed	Unstable

If untreated, 20–25% of patients are at risk to developing secondary osteoarthritis and may require total hip replacement early in life (7, 8). Therefore, early diagnosis and treatment are fundamental to preventing disability later in life (4, 9, 10).

The treatment aims to achieve a concentric reposition, retention, and maturation of the hip (6, 11). In patients with an early diagnosis within the first 6 months of life, treatment is essentially functional and involves the use of dynamic harnesses and orthoses (12, 13). All of them are made to keep the hip in flexion and abduction to reduce the hip and promote hip maturation (14, 15).

**FIGURE 1:**
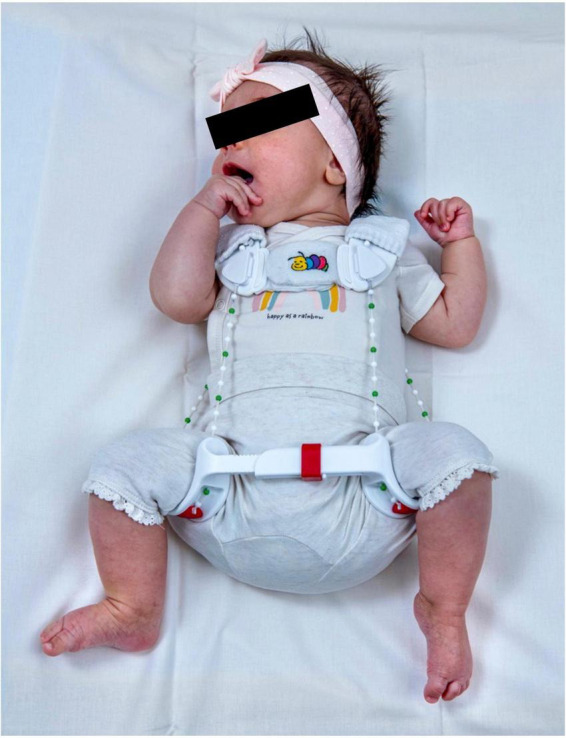
Tübingen splint.

The Tübingen splint, derived from the widely used Pavlik harness, is a rigid splint that maintains the flexion–position of the hip while limiting its abduction (16) (Figure 1).

Indicated harness positioning consists of 90°–110° of flexion and 45°–60° of abduction of both hips. This position is known as the safe zone to prevent tension on the capsular blood vessels and prevent avascular femoral head necrosis (AVN) (17). Initially, the device was conceived to treat DDH of types IIb and IIc designed as stable hips according to the Graf classification (Table 1) (18, 19). Later on, its use was also expanded to unstable DDH of grade D to grade IV with variable outcomes (19–22). The indication and treatment success have been confirmed by many authors for stable DDH, but there are still controversies in the literature regarding the treatment of unstable DDH with the Tübingen splint (23, 24). Some authors even recommend closed reduction and spica cast as the gold standard for unstable DDH (25).

Indeed, we believe that care should be taken when treating unstable DDH with the Tübingen splint. The aim of our study was to compare the outcome of stable and unstable DDH treated with the Tübingen splint in children aged 0–6 months.

## Materials and methods

After approval by the Ethics Review Committee (ID Number 2019-01761), we performed a monocentric retrospective study. We included all patients aged between 0 and 6 months treated for primary DDH with a Tübingen splint at our tertiary pediatric orthopedic center between May 2017 and February 2020.

All patients had ultrasonographic evaluation according to the Graf method and underwent dynamic clinical evaluation by applying the Barlow and Ortolani tests for initial diagnosis (19). All the ultrasounds were done by our pediatric radiologist.

Inclusion criteria were all children aged between 0 and 6 months at the moment of diagnosis, who had no other treatment before the Tübingen splint and who underwent a pelvic x-ray around 12 months of age.

Developmental dysplasia of the hip in association with neurodevelopmental disorders and patients treated with another device before the introduction of the Tübingen splint were excluded.

The treatment indication was based on the International Interdisciplinary Consensus Meeting on the Evaluation of Developmental Dysplasia of the Hip (5).

Patients with stable DDH of grades IIa, IIb, and IIc were required the splint at least 22 h a day. Remove of the splint was allowed for bath and diaper changing. Hip ultrasonography according to the Graf method was repeated every 6 weeks until normalization of the alpha angle.

Patients of parents with unstable DDH of grades D, III, and IV were trained to use the Tübingen splint continuously 24/24 h a day, with close ultrasonographic and clinical follow-up every 2–3 weeks. The treatment was then continued as mentioned above if the ultrasound showed improvement of DDH. Conversely, in the case of worsening of the alpha angle seen on ultrasound, we proceeded to a closed reduction and application of a spica cast under general anesthesia. Hip arthrography was done at the same time.

Patients in both groups underwent radiological follow-up by a plain pelvic x-ray between 12 and 24 months of age. The radiographies were done in a supine position with extended legs.

To compare the outcomes of both groups, we recorded the following parameters: (1) the degree of DDH according to the Graf classification at initial diagnosis, (2) age at treatment initiation, (3) duration of treatment, and (4) acetabular index measured on pelvic x-ray performed at the latest follow-up between 12 and 24 months of age.

Complications, especially avascular necrosis, were also recorded. The presence of AVN was determined based on the Kalamchi and McEwan classification (26). Risk factors have been recorded for general interest.

Successful treatment by the Tübingen splint was defined as a normal acetabular index measured on pelvic x-ray at last follow-up for hips treated only by the Tübingen splint. Acetabular index values were based on the normal percentile reference curves according to Novais et al. (27).

Unstable hips requiring closed reduction due to worsening of DDH seen on follow-up ultrasound after placement of the Tübingen splint were considered as failure, as well as those hips treated with the Tübingen splint alone showing residual hip dysplasia at last follow-up.

### Statistical analysis

We performed a descriptive statistical analysis to compare stable (grades IIa–IIc) and unstable hips (grades D–IV) using the Chi-Square test and Student’s *t*-test.

The Student’s *t*-test has been used to highlight the difference in treatment initiation timing between the two groups (stable vs. unstable hips). The Chi square test has been used to highlight the differences in treatment success.

Data were reported as a mean with SD.

## Results

A total of 45 patients (57 hips) met our inclusion criteria. Of them 39 (87%) patients were girls. The left hip was affected in 37 cases (65%). Hips were bilaterally abnormal in 13 patients (12 girls and one boy).

Looking at patients’ demographics, 42 hips (74%) presented stable DDH of grades IIa–IIc and 15 hips (26%) presented unstable DDH of grades D–IV according to the Graf classification.

Treatment was started on average at 56 (SD ± 38) days of life. The earliest time of diagnosis was at 3 days of age and the latest at 208 days (6 months). When regarding the time at the initiation of treatment, we observed a significant difference (*p* < 0.003) between stable and unstable hips with an average age at the start of treatment before 6 weeks of life in only 15% (7 hips) of stable hips and in 66% (10 hips) of unstable hips. Treatment initiation after the third month of life was seen in a total of 14 hips out of 57 (25%) with 13 stable hips and only one unstable hip (*p* < 0.0001).

The overall average duration of treatment with the Tübingen splint, when excluding those hips that required a treatment modification by closed reduction and spica cast under general anesthesia, was 16 weeks (SD ± 7). The mean age at the last follow-up was 14 months (SD ± 4). The mean duration of follow-up was 16 months (SD ± 5).

The demographics and characteristics of our study population can be found in Table 2.

**TABLE 2:** Global characteristics of our study population.

Treated patients	45
Number of Hips	57
Change for spica cast	6 patients (8 hips)
Bilaterality	13 (29%)
Female	39 (87%)
Mean age at treatment initiation (days)	56 (±38; min 3, max 208)
Mean duration of treatment excluding those hips that had spica cast (weeks)	16 (±7; min 10, max 29)
Mean follow-up	14 (±4) months
Risk factors	
Breech presentation	23 (29 hips)
Familiarity	12 (16 hips)
First born	13 (18 hips)
Graf classification	
Type IIa^–^	7 hips
Type IIb	9 hips
Type IIc	26 hips
Type D	5 hips
Type III	6 hips
Type IV	4 hips

Overall treatment success was seen in 45 hips (79%) including 39 stable hips and 6 unstable or dislocated hips.

Treatment failure occurred in 12 hips (21%) including 9 unstable hips. A total of 8 hips (4 hips type D, 2 hips type III, and 2 hips type IV) underwent a closed reduction and application of a spica cast because of worsening of DDH seen on ultrasound 2–3 weeks after treatment initiation by the Tübingen splint. The 4 other hips (1 hip type IIb, 2 hips type IIc, and 1 hip type IV) treated by the Tübingen splint alone presented residual hip dysplasia at the latest follow-up. For all the 3 stable hips presenting residual hip dysplasia, initiation of treatment occurred after 3 months of life.

The mean acetabular index in these 4 cases was 30° (min 29, max 31). There was a significant difference in an acetabular index comparing hips with a successful treatment (23° ± 4) and hips presenting residual hip dysplasia (30° ± 1; *p*-value 0.000006). No significant difference was found concerning the age at treatment initiation. However, we observed a statistically significant difference in the duration of treatment when comparing those hips with residual hip dysplasia (13 weeks ± 1) to those hips with normal values on the latest follow-up x-ray (16 weeks ± 7; *p*-value 0.006).

Looking at the groups of stable and unstable hips separately, the success rate was 93% (39 hips out of 42) and 40% (6 hips out of 15), respectively. Statistical analysis confirmed a significant difference (*p* < 0.001) in treatment success when comparing stable and unstable DDHs (Table 3).

**TABLE 3:** Treatment success and failures according to severity of DDH according to Graf classification (19).

Graf classification/Treatment outcome	IIa^–^	IIb	IIc	D	III	IV	Stable hips	Unstable hips
Success (*n* = 45)	6	9	24	1	4	1	39	6
Failure (*n* = 12)	0	1	2	4	2	3	3	9
Total (*N* = 57)	6	10	26	5	6	4	42	15

p-value stable vs. unstable < 0.001.

Complications were reported in 3 (5%) cases. One femoral nerve palsy (type IIc hip) occurred 5 days after treatment initiation and recovered 4 days after splint removal. The treatment could then be pursued with our standard protocol without further nerve palsy. We observed two partial femoral avascular head necrosis of type I according to the classification of Kalamchi and Mc Ewan on the pelvic x-ray at the last follow-up. Both patients presented with unstable DDH of grade D and grade III, respectively.

Regarding the 8 hips (6 patients) that underwent a treatment adaptation with closed reduction and spica cast, 2 hips needed an open reduction because of failure of treatment and irreducible hip; one hip presented residual hip dysplasia and 5 hips had normal acetabular indexes at the latest follow-up.

No statistical difference was found concerning the age at the beginning of treatment when compared to those hips treated solely by the Tübingen splint.

## Discussion

The overall treatment success rate with the Tübingen splint in our cohort was 79%. Stable and unstable DDH have a significant difference with a success rate of 93% and 40%, respectively, which is like previously published data in the literature (22, 24).

This significant difference in treatment success between stable and unstable hips has already been described by other authors. Ran et al. compared the Tübingen splint to the widely used Pavlik Harness. Successful treatment for unstable hips treated with the Tübingen splint was significantly lower than for the Pavlik Harness, especially in bilateral dislocation or in severe cases of hip dislocation as grade IV hips according to the Graf classification. Of the 43 hips treated with the Tübingen splint in their cohort, 14 hips (33%) had a poor outcome. Of them, 11 hips (79%) presented DDH of grade IV. The overall success rate was 67%, which is comparable to our results (23).

Other authors reported even failure rates as high as 78.6% for hips Graf grade IV in patients treated by a hip abduction flexion splint (28).

Our results support the treatment by closed reduction and spica casting as the gold standard for unstable hips, as already confirmed by other authors (25).

Other authors demonstrate higher success rates (29–32). Table 4 summarizes the data found in the literature.

**TABLE 4:** Review of literature.

Author	Country	Year	N° of hips	Treatment initiation (weeks)	Treatment duration (weeks)	Successful results	Screening program
Seidl et al. (21)	Germany	2012	50	1	7	98%	Universal
Munkhuu et al. (29)	Mongolia	2013	781	1	n.r.	100%	Universal
Atalar et al. (43)	Turkey	2014	60	18	17	93%	Selective
Pavone et al. (30)	Italy	2015	544	5	16	90%	Universal
Yegen et al. (50)	Turkey	2018	104	12	n.r.	75%	Selective
Ran et al. (23)	France	2020	43	14	17	67%	Selective
Kubo et al. (32)	Germany	2020	109	n.r.	n.r.	95%	Universal
Zhou et al. (24)	China	2020	203	9	18	84%	Selective
***Present study***	***Switzerland French part***	***2021***	***57***	***8***	***15***	***79%***	***Selective***

n.r: not reported.

These differences may be due to different factors; first, we could observe a different distribution of patients in some studies as seen in the cohorts from Pavone et al. and Seidl et al. They reported a 2.2% and a 2% rate of unstable hips, respectively, whereas the frequency of unstable hips was 17% in our series (30, 31).

Second, the lack of a universal screening program and the later referral of patients to our center with later treatment initiation is known as a negative predicting factor (33).

Indeed, overall initial treatment was established for an average of 56 days, which corresponds to 8 weeks of life. This is, compared to other authors, quite belated (29–31), especially if we admit that the highest post-maturation of the acetabulum after birth takes place in the first 16 weeks after birth (34).

Furthermore, failures in stable hips in our cohort concerned those patients in whom treatment was started after 3 months of life. This confirms that the earlier the treatment, the higher the success rates of treatment (29, 34, 35).

The significant difference between the start of treatment comparing stable and unstable hips in our study can be explained by the fact that stable hips are often detected late due to the absence of instability on clinical examination and therefore screening ultrasound of the hip is done later in life. This is even more evident when we look at the 14 hips in our study with treatment initiation after 3 months of life. Indeed, out of the 14 hips, which represents 25% of the hips in our series, only one hip was an unstable hip. These findings support that hip ultrasound is the only way to detect DDH, especially stable ones, with certainty (29).

It is one of the reasons why the international interdisciplinary consensus meeting on the evaluation of DDH in 2018 as well as many other authors support a universal screening program for DDH (5, 11, 36–39).

However, numerous recent studies and meta-analyses have not demonstrated the utility of universal screening in diminishing the incidence of late dysplasia (40, 41). Furthermore, Laborie et al. concluded that universal screening favors overtreatment (but not an increased rate of complications) while no significant reduction of late dysplasia was observed in comparison with selective screening (42).

We had to change the treatment in 14% (8 hips) of cases. All of them presented unstable hips according to Graf’s classification. This is in line with the results of other authors (43).

The average treatment time in our cohort was 3 months as already described in the literature (17, 30, 43).

Because the alpha angle shows a maturation of about one degree per week and the interobserver measurement error for the alpha angle at ultrasound is about 4–5 degrees, we repeated the ultrasound every 6 weeks until normalization of the alpha angle for stable hips (34, 44, 45). Regarding the unstable hips, it seemed important to us to carry out a close follow-up in order not to miss any worsening of the dysplasia which was an indication for a closed reduction and spica cast, a treatment considered as the gold standard by some authors (25).

The most frequent complication in our study was AVN of the femoral head in 5% (2 hips), which varies from 0 to 28% according to studies reported in the literature (46). However, our data may be an underestimation of the real number of AVNs given our short follow-up time of an average of 14 months.

Looking at the demographics of our study population, 87% of patients were girls. The latest epidemiological data reported that DDH is two to three times more common in females than in male infants (47, 48). A total of 77% of patients presented a positive family history or breech position. Both are known to be risk factors for DDH with a relative risk for the breech presentation of 3.8 (95% CI 2.3–6.2) and 1.39 (95% CI 1.23–1.57) for positive family history (48, 49). Nevertheless, there was no correlation between a specific risk factor and the observed failure rate.

The lack of information on whether parents removed the splint more than allowed by the caregivers, can be considered as a weak point of our study, as parental non-compliance seems to be one of the main reasons for the failure of the treatment (17).

The author also recognizes the small sample size of unstable hips (only 15 out of 57 hips) as a major limitation of the present analysis.

The strength of our study is that we were able to highlight the limitations of treatment with the Tübingen splint for unstable hips. The study supports the treatment by closed reduction and spica cast as the gold standard for unstable hips and the importance of close monitoring of unstable hips if treated with the Tübingen splint in order to be able to quickly change the treatment in the event of non-improvement.

## Conclusion

Our study confirms that the Tübingen splint is a safe and effective treatment for stable hips. For unstable hips in which treatment with a Tübingen splint is initiated, very close monitoring is mandatory in order to adapt the treatment in the event of poor evolution. The treatment of choice will then be closed reduction and spica cast.

## Data availability statement

The raw data supporting the conclusions of this article will be made available by the authors, without undue reservation.

## Ethics statement

The studies involving human participants were reviewed and approved by Commission Éthique du Canton de Vaud, Switzerland. Written informed consent to participate in this study was provided by the participants’ legal guardian/next of kin. Written informed consent was obtained from the individuals’ legal guardian/next of kin for the publication of any identifiable images or data included in this article.

## Author contributions

EC, C-AS, and SM: data curation. C-AS and SM: formal analysis. SM: methodology and supervision. P-YZ: project administration. ES: validation. EC: writing—original draft. ES, P-YZ, and SM: writing—review and editing. All authors contributed to the article and approved the submitted version.
