# Erratum: Comparison of treatment outcomes of stable and unstable developmental dysplasia of the hip with the Tübingen splint

**DOI:** 10.3389/fped.2023.1178331

**Published:** 2023-03-21

**Authors:** Frontiers Production Office

**Affiliations:** Frontiers Media SA, Lausanne, Switzerland

**Keywords:** DDH, graf method, spica cast, closed reduction, Tübingen splint, hip ultrasound

An Erratum on Comparison of treatment outcomes of stable and unstable developmental dysplasia of the hip with the Tübingen splint By Chaibi E, Saugy C-A, Samara E, Zambelli P-Y and Merckaert SR. (2022) Front. Pediatr. 10:976367. doi: 10.3389/fped.2022.976367

An omission to the funding section of the original article was made in error. The following sentence has been added: “Open access funding was provided by the University of Lausanne”.

The original version of this article has been updated.

